# Risk factors associated with pulmonary hypertension in patients with active tuberculosis and tuberculous destroyed lung: a retrospective study

**DOI:** 10.1038/s41598-024-59679-z

**Published:** 2024-05-02

**Authors:** Weijian Liu, Yuxiang Xu, Liangzi Yang, Senlin Zhan, Kaihua Pang, Hao Lin, Hongjuan Qin, Peize Zhang

**Affiliations:** Department of Pulmonary Medicine and Tuberculosis, The Third People’s Hospital of Shenzhen, National Clinical Research Center for Infectious Disease, Southern University of Science and Technology, Shenzhen, China

**Keywords:** Pulmonary tuberculosis (PTB), Tuberculous destructive lung (TDL), Pulmonary hypertension (PH), Aging, D-dimer, Tuberculosis, Respiratory tract diseases

## Abstract

Pulmonary tuberculosis (TB) can result in irreversible damage and lead to tuberculous destructive lung (TDL), a severe chronic lung disease that is associated with a high mortality rate. Additionally, pulmonary hypertension (PH) is a hemodynamic disorder that can be caused by lung diseases. The objective of this study is to investigate the risk factors associated with PH in active TB patients diagnosed with TDL. We conducted a retrospective review of the medical records of 237 patients who were diagnosed with TDL, active pulmonary tuberculosis, and underwent echocardiography at the Third People’ Hospital of Shenzhen from January 1, 2016, to June 30, 2023. Univariate and multivariate logistic regression analyses were performed to identify factors that correlated with the development of pulmonary hypertension. Univariate and multivariate logistic regression analyses revealed that several factors were associated with an increased risk of pulmonary hypertension (PH) in individuals with tuberculosis destroyed lung (TDL). These factors included age (OR = 1.055), dyspnea (OR = 10.728), D-dimer (OR = 1.27), PaCO2 (OR = 1.040), number of destroyed lung lobes (OR = 5.584), bronchiectasis (OR = 3.205), and chronic pleuritis (OR = 2.841). When age, D-dimer, PaCO2, and number of destroyed lung lobes were combined, the predictive value for PH in patients with TDL was found to be 80.6% (95% CI 0.739–0.873),with a sensitivity of 76.6% and specificity of 73.2%. Advanced age, elevated D-dimer levels, hypercapnia, and severe lung damage were strongly correlated with the onset of PH in individuals with active pulmonary tuberculosis (PTB) and TDL. Furthermore, a model incorporating age, D-dimer, PaCO2, and the number of destroyed lung lobes might be valuable in predicting the occurrence of PH in patients with active PTB and TDL.

## Introduction

Tuberculosis (TB) remains a significant global public health threat, with 10.6 million new cases and 1.6 million deaths reported in 2021^1^. Among the different types of TB, pulmonary TB is the most commonly observed, and it can cause irreversible damage to the lung parenchyma, bronchi, and lymph nodes, leading to a condition known as tuberculous destructive lung (TDL)^2^. TDL is characterized by a decrease in lung and airway volume, progressive airflow limitation, and recurrent exacerbations. It is a severe complication of pulmonary TB and is associated with high mortality^3^. Pulmonary hypertension (PH) is a hemodynamic disorder defined by a mean pulmonary arterial pressure of 25 mm Hg (1 mmHg = 0.133 kPa) or higher at rest during the Right-sided Heart Catheterization (RHC). Non-invasive transthoracic echocardiography is commonly used clinically to estimate the probability of PH^4^. PH due to chronic lung diseases is classified as Group 3 according to clinical practice guidelines^5^. Although TB is not commonly recognized as a cause of PH, previous studies have demonstrated the onset of PH in TB survivors^6,7^. As one of the complications of TDL, PH frequently complicates the clinical course of lung disease, reduces functional ability, impairs quality of life, causes right ventricular dysfunction and heart failure, thereby worsening the prognosis of patients with TDL^8–10^. However, the prevalence and clinical implications of PH in active pulmonary TB patients with TDL have not been well understood, and the risk factors for the development of PH have not been clearly identified^11^. Therefore, in this study, we aim to analyze clinical data, imaging characteristics, and echocardiography findings to explore the risk factors for PH in active TB patients with TDL.

## Methods

### Methods and materials

#### Study design and participants

**Figure 1:**
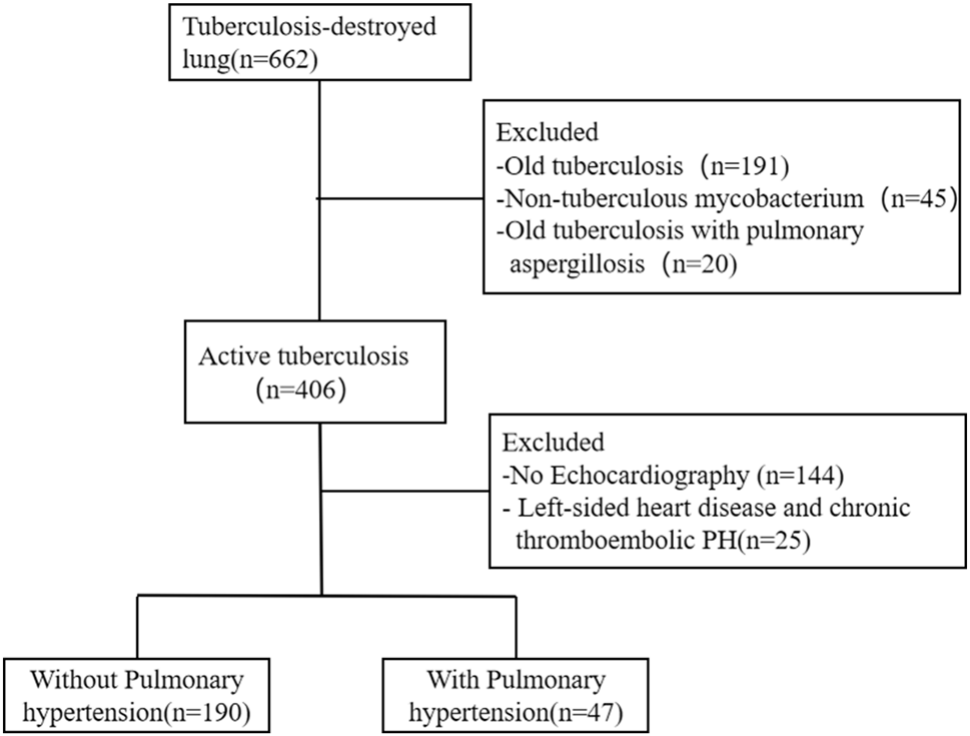
Flow diagram of the study.

We conducted a retrospective study to investigate the relationship between tuberculous destructive lung (TDL) and pulmonary hypertension (PH). We reviewed the medical records of 662 patients who were diagnosed with active pulmonary TB and TDL at the Third People’s Hospital of Shenzhen from January 1, 2016, to June 30, 2023. The inclusion and exclusion criteria were as follows: (1) Inclusion criteria: active tuberculosis and TDL patients who underwent echocardiography. (2) Exclusion criteria: non-active pulmonary TB; non-tuberculous mycobacterial lung diseases; PH associated with left heart diseases, chronic obstructive lung diseases or emphysema, restrictive lung disease, connective tissue disease, chronic pulmonary embolism, pulmonary artery obstructions and idiopathic PH. Among them, we excluded 191 cases of non-active pulmonary tuberculosis, 20 cases of old pulmonary tuberculosis complicated with aspergillosis, and 45 cases of non-tuberculous mycobacterial lung disease. We included 406 cases of active pulmonary tuberculosis complicated with TDL. Furthermore, we excluded 144 cases with no echocardiography and 25 cases with PH caused by left-sided heart diseases and chronic thromboembolic PH. Ultimately, we included 237 cases in the analysis (Fig. 1). This study was approved by the Ethical Committee of the Third People’ Hospital of Shenzhen (IRB number: 2021-014-02). Written informed consent of patients was waived by the ethics committee (Ethical Committee of the Third People’ Hospital of Shenzhen) as all clinical data were extracted from the medical system and personal information of any patient was masked. The hospital ensured that no personal information of any patients was involved in using these data, and they adhered to the Declaration of Helsinki regarding confidentiality and ethical standards.

#### Definitions

Active pulmonary tuberculosis is defined according to the diagnostic criteria issued by the World Health Organization^12^. The detailed diagnosis criteria of active tuberculosis were as below: ① tuberculosis pathogenicity testing (positive fast-acid bacilli smear or culture results or molecular test) ② a comprehensive assessment based on the patient's clinical manifestations, laboratory testing, and radiological findings.

Tuberculosis destroyed lung (TDL) refers to extensive structural damage of the lung caused by Mycobacterium tuberculosis infection. This damage is manifested by widespread bronchial distortion, bronchiectasis, pulmonary fibrosis, cavitation, caseous necrosis, lymph node necrosis, and lymphatic obstruction, resulting in the loss of normal lung function. The assessment of TDL was conducted by one radiologist and one pulmonary tuberculosis specialist. In case of disagreement, another senior radiologist was consulted. Doppler echocardiography was used in our study to estimate the pulmonary artery systolic pressure (PASP) and assess the presence of pulmonary hypertension (PH) in the patients. A PASP measurement above 35 mmHg indicated PH in our study^4^. Right atrium (RA) pressure was also used in the determination of PASP. Estimation of RA pressure is based on inferior vena cava (IVC) size and collapse. IVC diameter > 2.1 cm that collapses < 50% with a sniff suggests high RA pressure of 15 mm Hg, which indicates the onset of PH^13,14^.

#### Data collection

For all eligible patients, the recorded information included demographics, medical history, underlying comorbidities, laboratory findings, Chest CT images, and cardiac ultrasound reports were obtained. Laboratory findings included white blood cell (WBC) count, neutrophilic granulocyte (GRA), lymphocyte (LYN), hemoglobin (HGB), monocyte (MONO), C-reactive protein (CRP), procalcitonin (PCT), D-Dimer, liver, and kidney function. All these clinical data were within 1 week of admission. A trained team of physicians and imaging specialists at The Third People's Hospital of Shenzhen reviewed the Chest CT images and cardiac ultrasound reports. The occurrence of destructive lung, pleural effusion, bronchiectasis, and PH were documented.

#### Statistical analyses

All statistical analyses were conducted using SPSS 26.0 (IBM, USA). Normal distribution data were expressed as mean ± standard deviation (SD), while skewed distribution data were presented as median and quantile. The significance of continuous data was determined using either a student's t-test or a Mann Whitney U test. Comparisons between two groups were performed using either a One-way Anova or a K-W test. Categorical features were presented as frequency (%) and compared using the chi-squared test. Differences in non-normally distributed data between two groups were assessed using the Mann–Whitney U test. Univariate and multivariate linear regression analyses were performed to identify factors correlated with PH. The association of variables with pulmonary arterial hypertension was evaluated using the receiver operating characteristic curve (ROC) and the area under the curve (AUC). Statistical significance was defined as a p-value < 0.05, and all reported p-values were two-sided.

## Results

### Clinical characteristics between TDL patients with and without PH

According to the measurement of pulmonary artery systolic pressure (PASP) through Doppler echocardiography, a total of 237 patients with tuberculous destroyed lung (TDL) were divided into two groups: TDL with PH group (47 cases) and TDL without PH group (190 cases). The mean age of the enrolled patients was 50.5 ± 17.1 years. Within the TDL with PH group, the mean age was 58.5 ± 15.8 years, while within the TDL without PH group, the mean age was 48.5 ± 16.9 years. The difference in age between the two groups was statistically significant (*P* < 0.001). Among the patients, 59 females (24.9%) were identified, with 19 in the TDL with PH group and 46 in the TDL without PH group. There was no statistically significant difference in gender composition between the two groups. Moreover, no statistically significant differences were observed between the two groups of patients in terms of body mass index (BMI), duration of symptoms, smoking history, diabetes, malnutrition, treatment history, and drug resistance. However, the incidence of hypertension was higher in the TDL with PH group (14.9%) compared to the TDL without PH group (5.3%), and this difference was statistically significant (*P* = 0.022). Regarding clinical symptoms, patients in the TDL with PH group had a higher incidence of dyspnea and bilateral lower extremity edema compared to the TDL without PH group (93.6% vs. 66.8%, P < 0.001; 19.1% vs. 8.4%, P = 0.032, respectively). However, there were no significant differences between the two groups in terms of cough, sputum, and hemoptysis (*P* > 0.05). Furthermore, the partial pressure of carbon dioxide (PaCO2) in the TDL with PH group was higher than that in the TDL group (*P* = 0.001), while the arterial/alveolar oxygen ratio was lower in the TDL with PH group compared to the TDL without PH group (*P* = 0.005). Both differences showed statistical significance (Table 1).

**Table 1:** Comparison of clinical characteristics of TDL patients with and without PH.

	TDL patients with PH (n = 47)	TDL patients without PH (n = 190)	Total (n = 237)	*p*-value
Age (years), mean ± SD	58.5 ± 15.8	48.5 ± 16.9	50.5 ± 17.1	< 0.001
Gender (female, %)	13 (27.7%)	46 (24.2%)	59 (24.9%)	0.624^#^
Body mass index (kg/m^2^)	17.57 (14.88, 20.42)	17.63 (15.25, 19.49)	17.63 (15.10, 19.55)	0.999*
Duration of symptoms (months)	2.0 (11.0, 48.0)	6.0 (2.0, 36.0)	6.0 (2.0, 36.0)	0.505*
Smoking status (%)	19 (40.4%)	84 (44.2%)	103 (43.5%)	0.639^#^
Comorbidities				
Diabetes mellitus (%)	14 (29.8%)	45 (23.7%)	49 (24.9%)	0.386^#^
Hypertension (%)	7 (14.9%)	10 (5.3%)	17 (7.2%)	0.022^#^
Malnutrition (%)	26 (55.3%)	123 (64.7%)	149 (62.9%)	0.232^#^
Previous irregular TB treatment (%)	12 (25.5%)	74 (38.9%)	86 (36.3%)	0.087^#^
Drug-suseptible tuberculosis (%)	40 (85.1%)	138 (72.6%)	178 (75.1%)	0.077^#^
Evidence for aetiology				
Culture positive	26 (61.9%)	127 (73.0%)	153 (70.8%)	0.156
AFB smear positive	25 (62.5%)	103 (62.0%)	128 (62.1%)	0.958
Molecular test positive	32 (18.5%)	141 (74.2%)	173 (73.0%)	0.397
Clinical manifestations				
Cough	46 (97.9%)	182 (95.8%)	228 (96.2%)	0.504^#^
Expectoration	46 (97.9%)	179 (94.2%)	225 (94.9%)	0.305^#^
Hemoptysis	7 (14.9%)	33 (17.4%)	40 (16.9%)	0.685^#^
Dyspnea	44 (93.6%)	127 (66.8%)	171 (72.2%)	< 0.001^#^
Lower limbs edema	9 (19.1%)	16 (8.4%)	25 (10.5%)	0.032^#^
Laboratory testing				
WBC count (× 10^9^/L)	8.15 (6.31, 10.50)	9.08 (6.64, 12.06)	8.94 (6.53, 11.96)	0.20*
Neutrophils ratio (%)	76.9 (71.8, 83.9)	78.20 (70.28, 85.70)	77.90 (70.85, 85.35)	0.525*
Lymphocyte ratio (%)	11.0 (6.7, 16.9)	11.8 (6.2, 17.4)	11.6 (6.4, 17.1)	0.909*
NRL	6.89 (4.13, 12.72)	6.46 (4.39, 13.75)	6.51 (4.38, 13.15)	0.816*
RBC count (× 10^9^/L)	4.05 ± 0.93	4.11 ± 0.73	4.09 ± 0.77	0.679
Haemoglobin (g/l)	109.47 ± 19.22	111.30 ± 22.26	110.94 ± 21.67	0.605
ALT (U/L)	20.0 (10.0, 32.6)	15.0 (8.8, 23.3)	15.8 (9.0, 25.4)	0.094*
AST (U/L)	29.0 (19.0, 43.0)	22.0 (15.9, 34.1)	22.4 (16.0, 35.1)	0.036*
Total bilirubin (μmol/L)	11.8 (6.4, 20.8)	8.9 (6.5, 13.1)	9.4 (6.5, 14.4)	0.046*
Albumin (g/L)	32.9 (29.9, 34.9)	34.0 (29.3, 38.6)	33.2 (29.5, 38.1)	0.248*
Serum creatinine (μmol/L)	65.0 (52.0, 78.0)	54.0 (45.9, 68.0)	56.0 (47.0, 70.0)	0.001*
Uric acid (μmol/L)	333 (241, 403)	284 (221, 352)	291 (225, 366)	0.045*
CRP (mg/L)	45.73 (17.87, 77.61)	65.97 (30.02, 113.91)	63.28 (25.34, 96.06)	0.02*
PCT (ng/ml)	0.12 (0.07, 0.44)	0.19 (0.07, 0.83)	0.17 (0.07, 0.69)	0.216*
ESR (mm/h)	65.0 (33.0, 87.0)	64.8 (40.8, 88.3)	64.8 (40.0, 80.0)	0.78*
D-dimer (μg/ml)	2.41 (1.42, 3.65)	1.32 (0.77, 2.57)	1.65 (0.86, 2.68)	< 0.001*
PaO_2_ (mm Hg)	87.9 (76.8, 100.0)	92.1 (74.2, 103.3)	91.5 (74.5, 102.5)	0.895*
PaCO_2_ (mm Hg)	46.2 (37.9, 54.6)	41.6 (36.2, 43.8)	41.9 (36.4, 45.5)	0.001*
PO2 (a/A) (T)	58.7 (47.4, 70.0)	66.7 (56.6, 84.5)	66.5 (51.9, 81.2)	0.004*
Echocardiography				
Ejection fraction (%)	63 (56, 67)	64 (60, 68)	63 (60, 68)	0.171
Radiological findings				
Number of destroyed lung lobe	2 (1, 2)	1 (1, 2)	1 (1, 2)	< 0.001*
Bronchiectasis	25 (53.2%)	59 (31.1%)	84 (35.4%)	0.004^#^
Pulmonary cavity	33 (70.2%)	133 (70.0%)	166 (70.0%)	0.977^#^
Chronic pleuritis	33 (70.2%)	83 (43.7%)	116 (48.9%)	0.001^#^
Atelectasis	6 (3.2%)	5 (10.5%)	11 (4.6%)	0.029
Emphysema	12 (6.3%)	7 (14.9%)	19 (8.0%)	0.053

*AFB* acid-fast bacilli, *WBC* white blood cell, *NRL* neutrophilto-lymphocyte ratio, *RBC* red blood cell, *Hb* haemoglobin, *ALT* alanine aminotransferase, *AST* aspartate aminotransferase, *CRP* C-reactive protein, *PCT* procalcitonin, *ESR* erythrocyte sedimentation rate, *PaO*_*2*_ arterial partial pressure of carbon dioxide, *PaCO*_*2*_ arterial partial pressure of carbon dioxide, *PO2(a/A)(T)* arterial oxygen/alveolar oxygen.

*Rank sum test; ^#^chi-square test; others for the independent samplet-tests.

### Imaging characteristics between TDL patients with and without PH

There was also no significant difference in cardiac ejection fraction between the two groups (*P* = 0.171). The median number of destroyed lung lobes on CT images in the TDL with PH group was 2, which was significantly different those in the TDL without PH group (*P* < 0.001). The incidence of bronchiectasis, pleural effusion, and atelectasis in the TDL with PH group were 53.2% vs. 31.1% (*P* = 0.004), 70.2% vs. 43.7% (*P* = 0.001), and 10.5% vs. 3.2% (*P* = 0.029), respectively, all with significant statistical differences.

### Univariate and logistic regression analysis of factors correlated with PH

Using pulmonary hypertension (0 = no, 1 = yes) as the dependent variable, binary logistic regression analysis was conducted with the indicators (age, dyspnea, d-dimer, Number of destroyed lung lobe, bronchiectasis, chronic pleuritis) that showed statistical significance in the univariate analysis serving as the independent variables. The results indicated that the above indicators had beta values greater than 0, which were independent risk factors of pulmonary hypertension in individuals with tuberculosis-related lung damage. What’s more, the higher the OR, the higher the hazard correlation coefficient. The study proved that risk factors included age (OR = 1.055), dyspnoea (OR = 10.728), D-dimer (OR = 1.27), PaCO2 (OR = 1.040), number of destroyed lobes (OR = 5.584), bronchiectasis (OR = 3.205) and chronic pleurisy (OR = 2.841). All of the above variables were statistically significant (Table 2).

**Table 2:** Factors correlated with PH in binary logistic regression analysis.

Variables	*B*	*p*-value	OR	95% CI	
				Lower limit	Upper limit
Age	0.054	< 0.001	1.055	1.025	1.086
Dyspnea	2.373	0.008	10.728	1.837	62.651
D-dimer	0.239	0.025	1.270	1.031	1.566
PaCO_2_	0.039	0.009	1.040	1.010	1.071
Number of destroyed lung lobe	1.720	< 0.001	5.584	2.237	13.942
Bronchiectasis	1.165	0.010	3.205	1.325	7.754
Chronic pleuritis	1.044	0.023	2.841	1.152	7.005

### Diagnostic efficacy of a prediction model for PH in patients with active PTB with TDL

**Figure 2:**
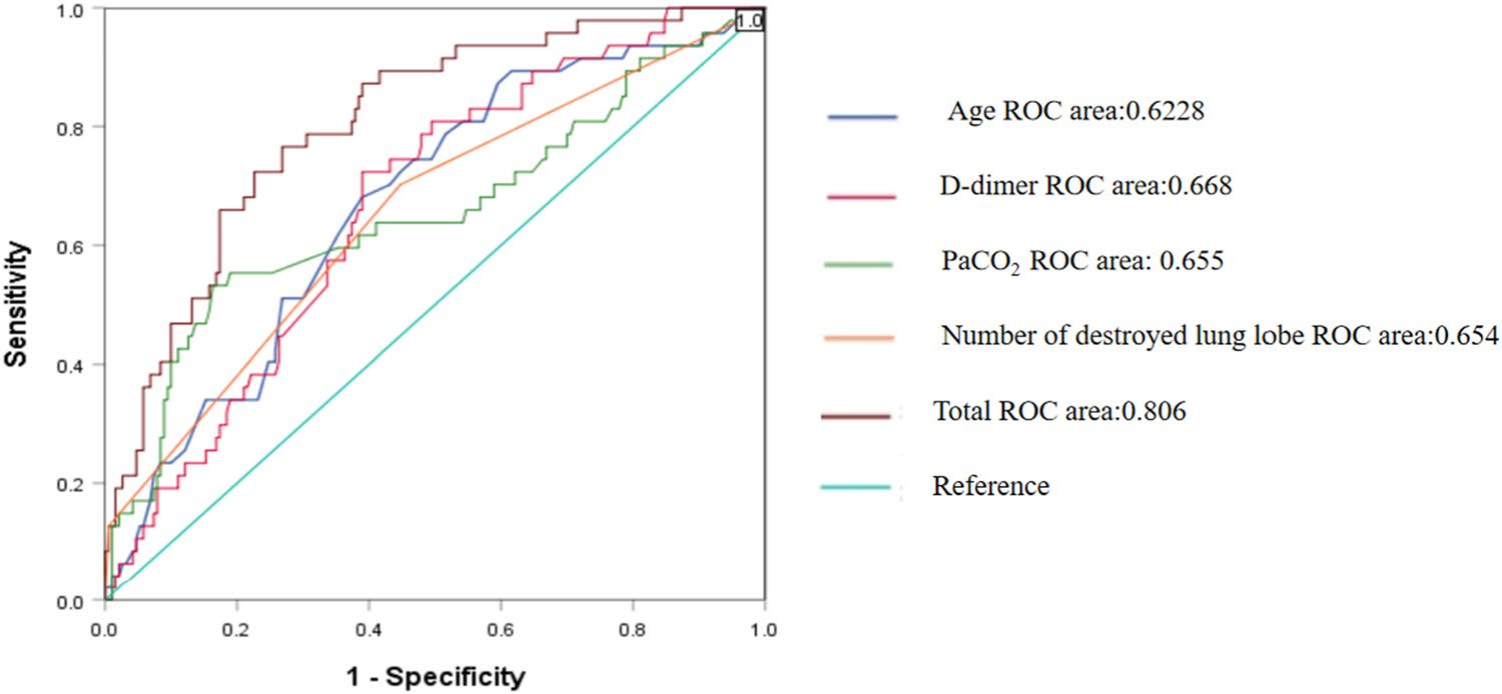
Receiver operator characteristic curve of the best model for predicting TDL with PH, as well as that of the individual.

The diagnostic efficacy of age, D-dimer, PaCO2,the number of destroyed lung lobes, and a combined model based on these four variables were evaluated for the diagnosis of pulmonary hypertension (PH) in patients with TDL.The diagnostic efficacy of the combined model based on these four variables for the diagnosis of pulmonary hypertension (PH) in patients with TDL was assessed using the cut-off value, sensitivity, specificity, Youden's index, and AUC, which was 0.806 (95% CI 0.805–0.871), with a sensitivity of 76.6% and a specificity of 73.2% (Table 3). The optimal cutoff point was 0.2, resulting in a Youden index of 0.498 (Fig. 2).

**Table 3:** Comparison of age, D-dimer, PaCO_2_, Number of destroyed lung lobe and the best model for predicting TDL with PH.

Variable	Cut-off	Sensitivity	Specificity	Youden	AUC	*P* value	95% CI	
							Lower limit	Upper limit
Age	53.5	0.681	0.661	0.292	0.671	< 0.001	0.587	0.754
D-dimer	1.865	0.723	0.611	0.334	0.668	< 0.001	0.589	0.747
PaCO_2_	46.05	0.532	0.837	0.369	0.655	0.001	0.558	0.753
Number of destroyed lung lobe	2.5	0.702	0.553	0.255	0.654	0.001	0.564	0.744
Best model	0.2	0.766	0.732	0.498	0.806	< 0.001	0.739	0.873

## Discussion

In our study, we demonstrated that active PTB patients with both TDL and PH were older and exhibited more severe clinical symptoms and extensive lung damage compared to those with TDL alone. Additionally, our study revealed that elevated levels of D-Dimer and hypercapnia were commonly observed in patients with both TDL and PH.

TDL mainly affected elderly patients, the mean age in this study was about 50 years old. TDL patients with PH were about ten years older than those without PH. This aligned with previous research indicating that older COPD patients are more likely to develop PH, implying that the prevalence of PH may increase with age in individuals with lung structural damage^15,16^. Furthermore, our study found that a higher proportion of patients with PH reported experiencing dyspnea compared to those without PH. While dyspnea is not a typical symptom of patients with PH, it can be indicative of impaired gas exchange. This highlights the importance of conducting cardiac ultrasounds and screening for the possible onset of PH in patients with TDL who present with dyspnea. Early diagnosis and management of PH may have the potential to enhance the quality of life and slow disease progression for these individuals.

In our study, we found out that patients with PH exhibited variable lung damage, including increased numbers of destroyed lung lobes, bronchiectasis, and chronic pleuritis. A retrospective study conducted in South Korea also revealed that patients with PH associated with tuberculous destroyed lung (TDL) experienced more severe lung damage^17^. TDL may lead to airflow limitation and the loss of the capillary network, thus contributing to the development of PH^18^. It has been observed in a study conducted by Li et al. that patients with active TB and elevated PASP frequently presented with lung cavities and extensive lung destruction. PaO2 levels were inversely correlated with PASP values in these patients^19^. But in our study, we didn’t found correlation between PaO2 level and PH. We contributed the possible reasons to different study population in two study. In our study, the enrolled patients were those with active PTB and TDL, while in Li’s study, the participants were all active PTB patients. Our study focused more on patients with severely damaged lung structures, who may basically have lower PaO2 level. However, the underlying pathophysiological mechanisms behind the development of PH in PTB patients remain unclear. Hypoxemia caused by chronic lung disease might be a contributor to PH, as it induces pulmonary vasoconstriction and increases PASP^20^. But recent studies have revealed that chronic hypercapnia, resulting from the direct effect of carbon dioxide on muscular pulmonary arteries, may also contribute to PH by causing recurring contraction and remodeling of pulmonary vessels. Hypercapnia is an important pathogenic factor in the development of PH^21^. In our study, we observed a correlation between severe lung destruction, elevated PaCO2 levels, and the development of PH in patients with TDL. We propose that the elevated PaCO2 levels are a result of impaired gas exchange due to lung destruction. Furthermore, the elevated PaCO2 levels contribute to increased vascular resistance, ultimately leading to PH.

Moreover, we observed that patients with TDL and PH had a higher level of D-dimer compared to patients with TDL alone. Univariate and logistic regression analyses revealed that elevated D-dimer was a risk factor for TDL with PH. This suggests that hypercoagulability may play a role in the development of PH^22^. Elevated levels of D-dimer, a byproduct of fibrin degradation, indicate a state of hypercoagulation in the body. Various inflammatory mediators, triggered by factors such as hypoxia, hypercapnia, infection, and others, can increase the production of fibrinogen and D-dimer. This creates a hypercoagulable state that can increase pulmonary artery pressure^23^. Previous studies have also reported higher levels of D-dimer in patients with pulmonary tuberculosis (PTB) and elevated PASP^19^. Additionally, recent research has demonstrated the benefits of oral anticoagulant therapy for patients with chronic obstructive pulmonary disease and PH^24^. Further investigations are required to provide more reliable evidence regarding the onset of a hypercoagulable state in patients with PH and TDL, as well as the potential benefits and safety of anticoagulants in this population.

For predicting PH in patients with TDL, we developed a model based on four variables: age, D-dimer levels, PaCO2 levels, and number of destroyed lung lobes. This model resulted in a sensitivity of 76.6% and specificity of 73.2%, the area under the curve (AUC) reached 80.6%. The combination of these four variables has a significant predictive value for PH in active PTB patients with TDL. Both tuberculosis (TB) and PH predominantly affect individuals in low-income and middle-income countries, millions of tuberculosis patients at risk of developing PH are unlikely to be identified and receive appropriate treatment due to the lack of skilled medical professionals and inadequate equipment^25^. Utilizing a simple yet effective predictive model for screening PH in active PTB patients with TDL might serve as an easily accessible assessment tool for early diagnosis. Further validation of our predictive model in an independent cohort study is needed.

There are several limitations in our study. Firstly, as a retrospective study, pulmonary function testing was not routinely performed in our study. We only used TDL on CT images to assess the damage of lung structures, which was insufficient for a comprehensive assessment of impaired lung function. Moreover, echocardiography was not a routine examination in all patients with TDL. The decision for echocardiography was up to the physician according to their personal experiences. This might introduce a selection bias and lead to missed diagnosis of PH. Secondly, we relied on Doppler echocardiography as the standard for assessing PH in our study. However, it is important to note that Doppler echocardiography is not considered the gold standard for diagnosing PH. Although previous studies have shown a strong correlation between echocardiography and right heart catheterization^26^, it is worth mentioning that echocardiography might underestimate the presence of PH^27^. Thirdly, it is worth mentioning that dyspnea, a symptom reported by the patients, was used as a diagnostic criterion in our study. It is important to recognize that subjective symptoms reported by patients may vary and have individual differences, thereby introducing potential selection bias.

In conclusion, our study has successfully demonstrated that several risk factors are correlated with the development of PH in active TB patients with TDL. These risk factors include old age, elevated levels of D-dimer, hypercapnia, and severe lung damage. By combining these four variables into a predictive model, we might be able to screen PH in active TB patients with severe lung damage. A well-designed prospective study is required to further investigate the exact contribution of PH and validate our predictive model in patients with severe tuberculous lung destruction.

## Data Availability

The datasets analyzed in the current study available from the corresponding author on reasonable request.
